# Maternal height and risk of caesarean section in singleton births in Sweden—A population-based study using data from the Swedish Pregnancy Register 2011 to 2016

**DOI:** 10.1371/journal.pone.0198124

**Published:** 2018-05-29

**Authors:** Ingrid Mogren, Maria Lindqvist, Kerstin Petersson, Carin Nilses, Rhonda Small, Gabriel Granåsen, Kristina Edvardsson

**Affiliations:** 1 Obstetrics and Gynecology, Department of Clinical Sciences, Umeå University, Umeå, Sweden; 2 Judith Lumley Centre, La Trobe University, Melbourne, Australia; 3 Department of Obstetrics and Gynecology, Västernorrland County Hospital, Sundsvall, Sweden; 4 Department of Women’s and Children’s Health, Division of Reproductive Health, Karolinska Institute, Stockholm, Sweden; 5 Department of Public Health and Clinical Medicine, Epidemiology and Global Health Unit, Umeå University, Umeå, Sweden; Helsingin Yliopisto, FINLAND

## Abstract

Caesarean section (CS) has short and long term adverse health consequences, and should therefore only be undertaken when necessary. Risk factors such as maternal age, maternal body mass index (BMI) and fetal weight have been extensively investigated in relation to CS, but the significance of maternal height has been less explored in Sweden. The aim was to investigate the significance of maternal height on risk of CS in a representative, population-based sample from Sweden, also taking into account confounders. Data on singleton births in the Swedish Pregnancy Register 2011 to 2016 were collected, including women with heights of 140 cm and above, constituting a sample of 581,844 women. Data were analysed with epidemiological and biostatistical methods. Mean height was 166.1 cm. Women born outside Sweden were significantly shorter than women born in Sweden (162.8 cm vs. 167.1 cm, p<0.001). There was a decreasing risk of CS with increasing maternal height. This effect remained after adjustment for other risk factors for CS such as maternal age, BMI, gestational age, parity, high birth weight and country of birth. Frequency of CS was higher among women born outside Sweden compared with Swedish-born women (17.3% vs. 16.0%), however, in a multiple regression model country of birth outside Sweden diminished as a risk factor for CS. Maternal height of 178–179 cm was associated with the lowest risk of CS (OR = 0.76, CI95% 0.71–0.81), whereas height below 160 cm explained 7% of CS cases. BMI and maternal age are established factors involved in clinical assessments related to birth, and maternal height should increasingly enjoy a similar status in these considerations. Moreover, when healthcare professionals are counselling pregnant women, taller stature should be more emphasized as a positive indicator for successful vaginal birth to increase pregnant women’s confidence in giving birth vaginally, with possible positive impacts for lowering CS rates.

## Introduction

Caesarean section (CS) is an operation used to reduce maternal and fetal complications of childbirth [1]. While it can be lifesaving for both the mother and the baby, CS is not without risks and should only be performed when indicated [1–3]. As caesarean sections may have both short and long term adverse health consequences for both the woman and the fetus/child, a CS rate as low as safely possible must be an overarching goal within obstetrics [1]. The number of CS has been increasing in both developed and developing countries, and while there may be medical reasons for this increase, non-medical factors may be partly responsible [1]. Short-term maternal complications of CS include anaesthesia-related complications [4], perioperative haemorrhage, infections, and thromboembolic disease [5], whereas for the child, respiratory distress is the primary health problem [6]. Significant long-term maternal complications include abdominal adhesions, bowel volvulus, infertility, abdominal pain, uterine rupture, placenta previa, and placenta accreta [7]. Children delivered by elective CS are more prone to allergy and asthma [8, 9]. In fact, one or more CS may result in a number of long-term adverse health outcomes in the child, both vaginal birth and caesarean section considered [10]. CS on request has become an increasing issue to manage over recent decades, and additionally there are rates worldwide that are clearly unmotivated from a medical point of view [5]. Further, rates differ greatly between countries [11].

A systematic review and meta-analysis of international migration and caesarean birth, concluded that caesarean rates between migrants and non-migrants differed in 69% of the studies, and there were consistently higher overall CS rates for Sub-Saharan African, Somali and South Asian women [12]. The increased rates for migrant women can probably be explained by a multiple of factors such as for example social and health determinants, communication barriers and cultural preferences [12]. A systematic review of ecological studies estimates that the optimal CS rate on a population level is 9% to 16%, and that at a level above this threshold there is no longer an association between reduced maternal and infant mortality and increasing CS rates [11].

Short adult height, which is an indicator of growth retardation, is a particular indicator of poor childhood nutrition in low and middle-income countries [13]. The variation in height within and across populations is also an indicator of the varying standard of living, nutrition, and biological deprivation that exists [13]. Sweden ranks number 17 for women and 15 for men among the world’s nationalities. The height difference between the tallest and shortest women (by nationalities) has remained at about 20 cm over the last century, even though adult height has changed significantly and unevenly across the world’s countries [14]. The countries with the tallest populations are located in Western Europe, and the shortest in Southeast Asia and Sub-Saharan Africa [13].

The influence of maternal height on obstetric and pregnancy outcomes has been identified in a number of studies across populations [13], with short stature showing an inverse association with a number of adverse pregnancy outcomes. In a study of 192,432 Swedish women, every cm decrease in maternal stature was associated with 0.2 days shortening of gestational age of the offspring, and increased the odds of preterm birth [15]. In a study of Danish women where short stature was identified as an obstetric risk factor, with higher prevalence rates of acute and elective caesarean sections, intra-uterine asphyxia, intra-uterine growth retardation and low Apgar-scores among infants of mothers who were less than 156 cm in height, compared with mothers who were 166–175 cm in height [16]. Another Nordic study of women from Denmark, Finland and Norway, investigating relationships between maternal height and fetal growth measures and gestational age, demonstrated that the relationship between maternal height and fetal birth weight and length is largely defined by fetal genetics, while the association with gestational age is more likely to be causal [17]. A systematic review and meta-analyses of the effect of maternal height on preterm birth and low birth weight (LBW) showed that short statured women had a higher risk of both preterm birth and LBW [18], associations that have also been reported in other studies [15, 18–23]. Maternal height has also been found to be inversely correlated with the risk of preeclampsia [24, 25], placental abruption [24], small for gestational age (SGA) [24], intrauterine growth restriction [22], and stillbirth [26]. Maternal height has shown associations with fetal growth patterns and birth weight [27], and has also been shown to be inversely correlated with the risk of caesarean delivery [22, 28–39], and a predictor of assisted delivery [22]. The possibility of predicting the likelihood of caesarean section by using the variables maternal age, height and assessment of infant birth weight has been suggested [30].

There has been a longstanding focus on risks during pregnancy and birth related to maternal body mass index (BMI), which is of great significance for clinical management, and pregnancy and childbirth outcomes [40]. Maternal height is clearly non-modifiable, but that is also the case with BMI during an ongoing pregnancy. The clinical significance of maternal height for outcomes of pregnancy and childbirth warrants increased clinical focus. Health professionals would likely benefit from having more evidence regarding maternal height and risk of CS in their counselling of pregnant women and in clinical management. An increasing number of pregnant women are requesting CS, which is an unwanted development from a medical perspective. This may be an overlooked opportunity for health professionals aiming at lowering the rate of unnecessary CS. This study aims to provide health professionals with comprehensive data about maternal height and caesarean section that can be easily used in the counselling situation. To our knowledge, no previous study has investigated this association in a population-based sample representative for contemporary Sweden. Ethical approval for this study was obtained from the Ethical Review Board in Umeå, Sweden (Dnr 2012-407-31M and Dnr 2014-152-32M).

## Aims

The overall aim was to investigate the significance of maternal height for risk of caesarean section in a representative, contemporary population-based sample from Sweden.

The specific aims were to examine *i*) risk of caesarean section in relation to maternal height, *ii)* risk of caesarean section in relation to maternal height with adjustment for other factors known to be associated with CS, including maternal country of birth.

## Methods

The Swedish Pregnancy Register was started in 2013 by merging the Maternal Health Care Register (established in 1999) [41, 42], and the National Quality Register for Prenatal Diagnosis (established in 2006) [43], and by initiation of collection of new information from deliveries [44]. Data from the Swedish Pregnancy Register (SPR) were retrieved for all women from 2011 to 2016 with information on pregnancy and delivery outcomes. The total primary sample included 591,754 pregnancies with a gestational age from 22 weeks + 0 days to 43 weeks + 0 days. After including only singleton pregnancies, i.e. excluding multiple births (1.7%), the final sample comprised 581,844 women. The distribution of births was 84,560 (14.5%; 2011), 93,077 (16.0%; 2012), 97,526 (16.8%; 2013), 96,524 (16.6%; 2014), 103,488 (17.8%; 2015) and 106,663 (18.3%; 2016) during the study period. During the period from 2011 to 2012 data were manually registered in SPR. From 2013, electronic transfer of data from digital medical records was initiated and the coverage of electronic transfer increased during the period from 2013 to 2016. The total number of births was compared with population data from Statistics Sweden (scb.se), a government body that collects information on *all* deliveries in Sweden and provides unique civic numbers to newborn children. The coverage for 2011 to 2016 was then estimated as 81%, 85%, 89%, 85%, 90% and 91%, respectively (personal communication). Information on maternal height was available for 570,515 women (98.1%). Women with height of 139 cm or less (n = 70) were excluded from the analysis, resulting in 570,445 women who could be included in analyses related to maternal height, corresponding to 98.0% of women in the singleton pregnancy sample.

### Explanatory and outcome variables

The key explanatory variable was maternal height (cm). Maternal height was used as a continuous variable and also categorized into 25 different 2 cm categories from 140 cm to 189 cm, with a last height category including maternal height from 190 cm to 198 cm. Other well-known explanatory variables were maternal age (years), body mass index (BMI; kg/m^2^), parity (primiparity, multiparity), birth weight (grams), gestational age (days), and country of birth (born in Sweden, born outside Sweden). Maternal age was calculated using the woman’s birth date and date of delivery. Body mass index was calculated from the equation maternal weight (kg) in early pregnancy divided by maternal height in metres squared (kg/m^2^). BMI was categorized in four classes according to the World Health Organization’s (WHO) classification; BMI <18.5 (underweight), BMI 18.5< 25.0 (normal weight), BMI 25.0< 30.0 (overweight), and BMI >30.0 (obesity) [45]. BMI was used as a continuous variable as well as a categorical variable in analyses. Birth weight was used as a continuous variable and also dichotomised in birth weight less than 4500 grams or 4500 grams or above (i.e. high birth weight). The main outcome variable of interest was caesarean section (CS), combining elective caesarean section and emergency caesarean section. Mode of delivery included the options vaginal delivery (including instrumental delivery), elective caesarean section, and emergency caesarean section. Preterm birth was defined as gestational age 154–258 days, term birth 259–293 days, and post-term birth 294–301 days in accordance with WHO recommendations endorsed in 1977. Some variables acted both as explanatory and as outcome variables.

### Statistics

Descriptive statistics present proportions and mean values. Pearson’s correlation coefficient was calculated to evaluate correlation between two variables. The independent samples t-test was used to test the difference between two mean values. One-way ANOVA was used to estimate difference between groups for continuous variables. Pearson’s Chi-Square test was used to test the relationship between categorical variables. Odds ratios (OR) and corresponding 95% confidence intervals (95% CI) were calculated using logistic regression analyses. The non-linear variable maternal height was modelled using a second degree polynomial in the multiple logistic regression analyses. The significance level was set at 0.05. Population attributable proportion (PAP)–that is the proportion of cases in the population attributable to the exposure–was calculated using the equation PAP = p(RR-1)/[1 + p(RR-1)] for specified exposures, where p = the proportion exposed in the population.

## Results

### Description of the data

Mean and median maternal heights for all women were 166.1 cm and 166.0 cm for the whole study period (Table 1). Mean heights for each year of the study period were 166.2 cm (2011), 166.2 cm (2012), 166.1 cm (2013), 166.2 cm (2014), 166.0 cm (2015), and 165.9 cm (2016). The overall proportions of women born in Sweden in relation to women born outside Sweden were 77.1% and 22.9% (Table 1). The corresponding proportions of births changed during the study period with increasing proportions of births to women born outside Sweden (p<0.001); 81.2%/18.2% (2011), 78.9%/21.1% (2012), 77.8%/22.2% (2013), 78.3%/21.7% (2014), 75.0%/25.0% (2015), and 71.3%/28.7% (2016). The distribution of primiparity/multiparity changed during the study period (p<0.001) with increasing proportions of multiparous women from 56.4% (2011) to 57.8% (2016). The overall mean maternal age was 30.75 years (Table 1) with a significant increase from 30.74 years in 2011 to 30.82 years in 2016 (p<0.001, one-way ANOVA). Mean BMI was 24.80 (kg/m^2^), with a significantly higher BMI in women born outside Sweden (25.03, p<0.001) in comparison to women born in Sweden (24.73; Table 1). BMI increased during the study period with the highest mean BMI in 2016 (24.91; p<0.001 one-way ANOVA). There was a negative correlation between increasing maternal height and BMI (r = -0.061; p<0.001). Maternal height, less than 150 cm (n = 2,570) and less than 156 cm (n = 30,056) constituted 0.45% and 5.3% respectively, of the total sample. T-test demonstrated a statistically significant difference in mean BMI between women with maternal height less than 150 cm compared to women with height 150 cm or above (25.55 vs. 24.80, p<0.001), and maternal height less than 156 cm in comparison with women with height 156 cm or above (25.46 vs. 24.77, p<0.001). Mean gestational age was 278.1 days and there was a positive correlation between increasing maternal height and increasing number of days of gestation (r = 0.055; p<0.001). There was a difference in gestational age where women born outside Sweden demonstrated a significantly shorter gestation in relation to women born in Sweden (Table 1). Mean birth weight was 3534 grams (g) and differed significantly between women born in Sweden (3565 g) and women born outside Sweden (3438 g; Table 1). During the study period there were significant changes in mean birth weights (p<0.001, one-way ANOVA): 3540 g (2011), 3543 g (2012), 3543 g (2013), 3528 g (2014), 3526 g (2015), and 3526 g (2016). Results on mode of delivery are presented in Table 1 where the majority of women (83.7%) gave birth vaginally whereas 16.3% gave birth through caesarean section. Women born outside Sweden were more likely to be delivered by CS; 17.3% of cases in comparison with 16.0% of women born in Sweden (p<0.001, Table 1). The annual rates of CS were 15.8% (2011), 15.8% (2012), 15.9% (2013), 16.7% (2014), 16.7% (2015) and 17.0% (2016).

**Table 1:** Background characteristics of women with singleton pregnancies (N = 581,844) and by maternal country of birth groups^a^^,^^b^.

Variables	All women	Women	Women	p-value^c^
	2011–2016 n (%)^d^	born in Sweden n (%)^e^	born outside Sweden n (%)^f^	
**Country of birth**	581,844 (100)			
Born in Sweden	423,564 (72.8)	423,564 (100)		
Born outside Sweden	125,930 (21.6)		125,930 (100)	
Missing values	32,350 (5.6)			
**Maternal height** (cm)	*570*,*445 (98*.*0)*	*417*,*518 (98*.*6)*	*124*,*255 (98*.*7)*	
Mean (SD^g^)	166.1 (6.5)	167.1 (6.1)	162.8 (6.6)	<0.001^a^
Min-Max	140–198	140–198	140–196	
**Maternal age (yrs)**	*581*,*675 (99*.*9%)*	*423*,*554 (99*.*9)*	*125*,*854 (99*.*9)*	
Mean (SD)	30.75 (5.2)	30.79 (5.1)	30.62 (5.5)	<0.001^a^
Min-Max	11.94–58.10	13.04–54.73	11.94–58.10	
**Maternal age in groups (years)**	*581*,*675 (99*.*9%)*	*423*,*554 (99*.*9)*	*125*,*854 (99*.*9)*	
≤19	7,404 (1.3)	4,413 (1.0)	2,247 (1.8)	<0.001^b^
20–24	75,639 (13.0)	53,029 (12.5)	18,375 (14.6)	
25–29	178,946 (30.8)	131,630 (31.1)	37,747 (30.0)	
30–34	194,516 (33.4)	144,455 (34.1)	39,461 (31.4)	
35–39	101,805 (17.5)	73,955 (17.5)	22,166 (17.6)	
≥40	23,365 (4.0)	16,072 (3.8)	5,858 (4.7)	
**Mode of delivery**	*581*,*817 (99*.*9)*	*423*,*541 (99*.*9)*	*125*,*926 (99*.*9)*	
Vaginal delivery	486,774 (83.7)	355,721 (84.0)	104,188 (82.7)	<0.001^b^
Caesarean section (CS)	95,943 (16.3)	67,820 (16.0)	21,738 (17.3)	
- Elective CS	41,102 (7.1)	29,765 (7.0)	8,922 (7.1)	<0.001^b^
- Emergency CS	53,941 (9.3)	38,055 (9.0)	12,816 (10.2)	
**Parity**	*573*,*569 (98*.*6)*	*418*,*950 (98*.*9)*	*125*,*193 (99*.*4)*	
Primiparous	245,914 (42.9)	188,069 (44.9)	47,625 (38.0)	<0.001^b^
Multiparous	327,655 (57.1)	230,881 (55.1)	77,568 (62.0)	
**Gestational age in days**	*571*,*882 (98*.*3)*	*416*,*385 (98*.*3)*	*123*,*375 (98*.*0)*	
Mean (SD)	278.1 (12.8)	278.3 (12.5)	277.8 (13.1)	<0.001^a^
Min-Max	154–301	154–301	154–301	
**Gestational age in groups**	*571*,*882 (98*.*3)*	*416*,*385 (98*.*3)*	*123*,*375 (98*.*0)*	
Pre-term	25,665 (4.5)	18,293 (4.4)	5,550 (4.5)	0.079^b^
Term	511,093 (89.4)	372,624 (89.5)	110,133 (89.3)	
Post-term	35,124 (6.1)	25,468 (6.1)	7,692 (6.2)	
**Maternal weight** (kg)	*565*,*029 (97*.*1)*	*413*,*617 (97*.*6)*	*123*,*236 (97*.*9)*	
Mean (SD)	68.5 (13.7)	69.1 (13.7)	66.4 (13.4)	<0.001^a^
Min-Max	32–195	33–195	32–178	
**BMI** (kg/m^2^)	*564*,*231 (97*.*0)*	*413*,*323 (97*.*6)*	*122*,*944 (97*.*6)*	
Mean (SD)	24.80 (4.7)	24.73 (4.7)	25.03 (4.8)	<0.001^a^
Min-max	12.84–71.63	13.40–71.63	12.86–67.09	
**BMI in groups**	*564*,*231 (97*.*0)*	*413*,*323 (97*.*6*	*122*,*944 (97*.*6)*	
<18.5	14439 (2.6)	9,446 (2.3)	4,224 (3.4)	<0.001^b^
18.5–24.99	331,867 (58.8)	249,385 (60.3)	66,326 (53.9)	
25–29.99	52,149 (9.2)	101,685 (24.6)	34,722 (28.2)	
30–34.99	16,472 (2.9)	36,499 (8.8)	12,984 (10.6)	
35–39.99	5,661 (1.0)	12,079 (2.9)	3,534 (2.9)	
≥40		4,229 (1.0)	1,154 (0.9)	
**Birth weight** (grams)	*576*,*145*	*419*,*646*	*124*,*525*	
Mean (SD)	3534 (561)	3565 (557)	3438 (553)	<0.001^a^
Min-Max	300–6640	300–6640	300–6240	
**Birth weight in groups**	*576*,*145*	*419*,*646*	*124*,*525*	
<4500 grams	96.4	96.1	97.7	
≥4500 grams	3.6	3.9	2.3	
≥4800 grams	1.0	1.2	0.7	
≥5000 grams	0.4	0.5	0.3	

^a^Test of difference for continuous variables with T-test

^b^Test of difference for categorical variables Pearson’s Chi-Square test

^c^Statistical significance at p<0.05

^d^Denominator = all women 2011 to 2016

^e^Denominator = all women with information on country of birth = Sweden

^f^Denominator = all women with information on country of birth = outside Sweden

^g^SD = standard deviation

### Caesarean section in relation to maternal height, country of birth and body mass index

**Fig 1:**
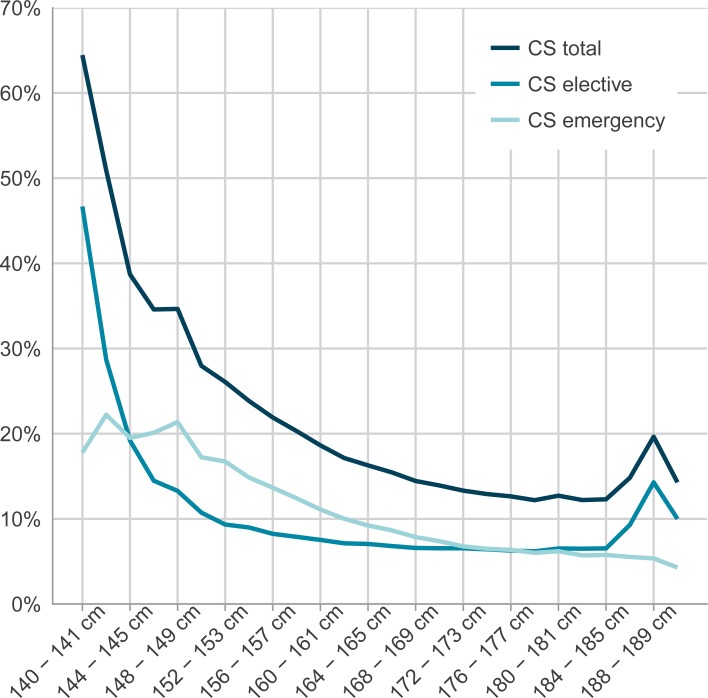
Frequency of caesarean sections (CS) total, elective CS and emergency CS for specified maternal height categories.

Maternal height distribution, rates of CS in relation to maternal height categories and country of birth are presented in Table 2 and Fig 1, where the overall lowest CS rates are noted among women with heights 178–179 cm and 182–183 cm (12.2%) and the highest rates of CS for short women. Women with height corresponding to the mean and median values (category 166–167 cm) demonstrated a CS rate of 15.5%, where increasing maternal stature demonstrated decreasing rates of CS with the exception of the tallest maternal heights (Table 2).

**Table 2:** Maternal height distribution, rates^1^^,^^2^ of caesarean section (CS) in relation to maternal height categories (N = 570,445) and country of birth.

Maternal height (cm)	All^1^		Born in Sweden^1^		Born outside Sweden^1^		CS^2^ %	Elective CS^2^ %	Emergency CS^2^ %	Born in Sweden^2^^,^^3^		Born outside Sweden^2^^,^^3^	
	n	%	n	%	n	%				Elective CS %	Emergency CS %	Elective CS %	Emergency CS %
**140–141**	45	0.01	9	0.002	32	0.03	64.4	46.7	17.8	55.6	11.1	43.8	21.9
**142–143**	108	0.02	28	0.005	75	0.04	50.9	28.7	22.2	25.0	21.4	28.0	21.3
**144–145**	323	0.06	68	0.02	235	0.2	38.7	19.2	19.5	20.6	27.9	18.3	18.3
**146–147**	746	0.08	194	0.05	487	0.4	34.6	14.5	20.1	21.1	19.1	11.7	21.1
**148–149**	1348	0.2	307	0.1	949	0.8	34.6	13.3	21.4	18.9	23.8	11.8	20.9
**150–151**	4799	0.8	1422	0.3	3039	2.4	28.0	10.7	17.2	12.9	19.3	9.7	16.5
**152–153**	8711	1.5	3334	0.8	4825	3.9	26.1	9.3	16.7	10.1	18.0	8.7	16.1
**154–155**	13976	2.5	5860	1.4	7181	5.8	23.8	9.0	14.9	10.0	16.1	8.3	13.9
**156–157**	21414	3.8	11218	2.7	8945	7.2	21.9	8.2	13.7	8.7	14.7	7.8	12.4
**158–159**	31501	5.5	18166	4.4	11545	9.3	20.3	7.9	12.4	8.3	12.8	7.3	11.9
**160–161**	52984	9.3	34139	8.2	15894	12.8	18.6	7.5	11.1	7.8	11.5	6.9	10.4
**162–163**	61920	10.9	43541	10.4	15157	12.2	17.1	7.1	10.1	7.3	10.4	6.6	9.3
**164–165**	74397	12.8	53809	12.9	16827	13.5	16.3	7.1	9.2	7.3	9.3	6.9	9.2
**166–167**	54502	9.4	42785	10.2	9095	7.3	15.5	6.8	8.6	6.9	8.7	6.4	8.1
**168–169**	66407	11.4	53271	12.8	10044	8.1	14.4	6.6	7.9	6.6	7.9	6.3	7.2
**170–171**	60186	10.3	49525	11.9	7887	6.3	13.9	6.5	7.4	6.5	7.4	6.3	7.2
**172–173**	44475	7.6	25905	9.0	5047	4.1	13.3	6.5	6.8	6.5	6.8	6.5	7.2
**174–175**	32056	5.5	18710	6.5	3419	2.8	12.9	6.4	6.5	6.4	6.4	5.9	7.1
**176–177**	16843	2.9	10059	3.5	1509	1.2	12.6	6.3	6.4	6.2	6.3	6.4	6.9
**178–179**	12269	2.1	7385	2.6	1042	0.8	12.2	6.2	6.0	6.1	5.9	6.4	7.2
**180–181**	7098	1.2	4352	1.5	618	0.5	12.7	6.5	6.2	6.6	6.4	5.2	5.3
**182–183**	2662	0.5	1568	0.5	247	0.2	12.2	6.5	5.7	6.6	6.0	6.5	3.6
**184–185**	1041	0.2	624	0.2	89	0.1	12.3	6.5	5.8	6.6	5.9	6.7	5.6
**186–187**	452	0.1	269	0.1	45	0.04	14.8	9.3	5.5	9.4	5.1	8.9	8.9
**188–189**	112	0.02	69	0.02	10	0.01	19.6	14.3	5.4	14.4	5.2	0	10.0
**≥190**	70	0.01	36	0.01	7	0.01	14.3	10.0	4.3	5.7	5.7	25.0	0
***Sum*: *n and/or %***	*570*,*445*	*100*	*417*,*518*	*100*	*124*,*255*	*100*	*n = 93*,*096* *100*	*n = 40*,*462* *7*.*1*	*n = 52*,*634* *9*.*2*	*n = 29*,*445* *7*.*1*	*n = 37*,*412* *9*.*0*	*n = 8*,*813* *7*.*1*	*n = 12*,*600* *10*.*1*

^1^Proportions calculated for the distribution in maternal height categories

^2^Proportions calculated for the distribution in each maternal height category

^3^Information on country of birth missing for n = 32,350

**Fig 2:**
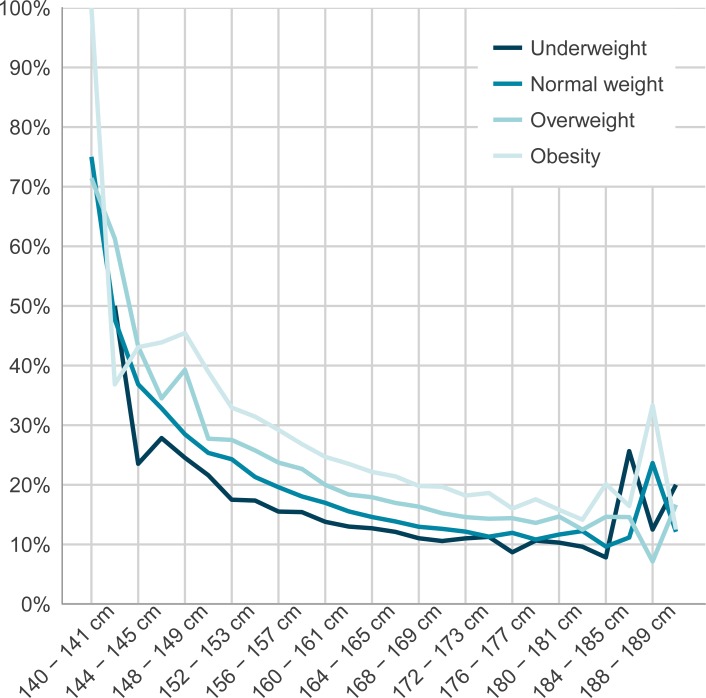
Frequencies of underweight, normal weight, overweight and obesity in relation to specified maternal heights categories.

BMI mean values in relation to maternal height and country of birth are presented in Table 3 where the lowest mean BMI of 24.2 is noted for the maternal height category of 178–179 cm. The overall rates of CS in relation to BMI-class were 12.0% (underweight), 14.3% (normal weight), 18.1% (overweight) and 22.6% (obesity). Rates of CS in relation to BMI-class for each maternal height category are presented in Table 4 and also in Fig 2. For each maternal height category there was an increased risk of CS in relation to increasing BMI-class with the exception of the tallest maternal height categories (Table 4, Fig 2).

**Table 3:** Body mass index (BMI) mean values in relation to maternal height and country of birth.

Maternal height (cm)	BMI		BMI range		BMI Born in Sweden		BMI Born outside Sweden	
	n	Mean value	Minimum	Maximum	n	Mean value	n	Mean value
**140–141**	44	24.99	18.11	37.76	9	26.70	31	24.66
**142–143**	106	25.38	17.12	43.00	27	25.90	74	25.21
**144–145**	320	25.92	15.91	43.40	68	26.62	232	25.24
**146–147**	736	25.33	16.20	44.10	193	25.72	480	25.24
**148–149**	1,331	25.62	15.31	48.65	305	26.11	936	25.42
**150–151**	4,754	25.56	15.40	54.38	1,412	25.97	3,013	25.32
**152–153**	8,643	25.45	14.28	67.09	3,312	25.55	4,787	25.33
**154–155**	13,831	25.42	13.49	52.71	5,816	25.37	7,100	25.48
**156–157**	21,216	25.25	13.97	62.88	11,126	25.16	8,874	25.37
**158–159**	31,173	25.06	14.42	56.17	17,991	24.97	11,429	25.19
**160–161**	52,404	25.16	14.27	65.62	33,811	25.11	15,721	25.25
**162–163**	61,286	24.95	14.10	65.87	43,123	24.91	14,997	25.08
**164–165**	73,630	24.90	12.86	71.63	53,309	24.88	16,658	24.97
**166–167**	53,912	24.70	13.40	64.18	42,359	24.67	8,982	24.86
**168–169**	65,646	24.65	13.82	59.88	52,715	24.63	9,922	24.66
**170–171**	59,503	24.60	14.19	62.98	49,021	24.59	7,787	24.71
**172–173**	43,967	24.43	12.84	56.13	37,018	24.43	5,001	24.43
**174–175**	31,692	24.46	13.54	58.12	26,919	24.45	3,383	26.64
**176–177**	16,649	24.37	15.00	51.98	14,438	24.36	1,493	24.59
**178–179**	12,129	24.22	13.89	51.76	10,578	24.23	1,033	24.38
**180–181**	7,003	24.49	15.43	56.48	6,073	24.50	612	24.32
**182–183**	2,621	24.56	15.10	48.30	2,265	24.55	246	24.45
**184–185**	1,025	24.70	15.10	44.90	898	24.71	87	24.68
**186–187**	447	25.40	17.34	44.32	388	25.66	44	23.70
**188–189**	111	24.36	17.64	38.76	96	24.14	10	23.83
**≥190**	70	24.99	15.10	37.20	53	25.24	12	24.74
***n;*** ***mean value***	*564*,*231*	*24*.*80*	*-*	*-*	*413*,*323*	*24*.*73*	*122*,*944*	*25*.*03*

**Table 4:** Rates of CS in relation to BMI-class for each maternal height category.

Maternal height (cm)	BMI-class	CS^1^ %	Elective CS^1^ %	Emergency^1^ CS^2^ %	Maternal height (cm)	BMI-class	CS^1^ %	Elective CS^1^ %	Emergency^1^ CS^2^ %
**140–141**	Underweight	0	0	0	**166–167**	Underweight	12.8	5.8	6.9
	Normal weight	57.1	47.6	9.5		Normal weight	13.6	6.3	7.3
	Overweight	71.4	28.6	42.9		Overweight	16.9	7.3	9.6
	Obesity	100	100	0		Obesity	21.4	8.4	13.1
**142–143**	Underweight	50.0	16.7	33.3	**168–169**	Underweight	10.8	5.7	5.1
	Normal weight	48.0	22.0	26.0		Normal weight	12.0	6.0	6.7
	Overweight	51.3	45.2	16.1		Overweight	16.3	7.3	9.1
	Obesity	36.8	15.8	21.1		Obesity	19.8	8.2	11.6
**144–145**	Underweight	22.2	0	22.2	**170–171**	Underweight	9.3	5.2	4.2
	Normal weight	34.8	17.1	17.7		Normal weight	12.4	6.1	6.4
	Overweight	43.7	19.5	24.1		Overweight	15.2	7.1	8.1
	Obesity	42.4	25.8	16.7		Obesity	19.4	7.9	11.7
**146–147**	Underweight	40.0	28.0	12.0	**172–173**	Underweight	10.5	5.4	5.1
	Normal weight	31.3	11.9	19.4		Normal weight	12.0	6.3	5.7
	Overweight	34.1	15.3	18.8		Overweight	14.6	6.7	7.8
	Obesity	43.9	18.7	25.2		Obesity	18.2	7.8	10.4
**148–149**	Underweight	30.3	15.2	15.2	**174–175**	Underweight	11.3	5.4	5.9
	Normal weight	27.7	11.2	16.5		Normal weight	11.3	5.8	5.4
	Overweight	39.3	15.6	23.7		Overweight	14.3	7.1	7.2
	Obesity	45.5	14.1	31.4		Obesity	18.6	7.9	10.7
**150–151**	Underweight	17.9	8.2	9.7	**176–177**	Underweight	9.4	6.6	2.8
	Normal weight	25.0	9.2	15.8		Normal weight	11.5	6.1	5.4
	Overweight	27.7	10.6	17.1		Overweight	14.3	6.4	7.9
	Obesity	38.9	15.8	23.1		Obesity	16.3	7.2	9.1
**152–153**	Underweight	16.9	5.9	11.0	**178–179**	Underweight	11.4	5.1	6.3
	Normal weight	23.5	8.1	15.3		Normal weight	10.8	5.6	5.2
	Overweight	27.5	10.0	17.5		Overweight	13.6	7.2	6.4
	Obesity	32.9	12.4	20.5		Obesity	17.5	7.6	9.9
**154–155**	Underweight	17.7	7.3	10.5	**180–181**	Underweight	9.2	3.3	5.8
	Normal weight	20.7	7.7	13.0		Normal weight	11.5	6.3	5.2
	Overweight	25.8	9.9	16.0		Overweight	14.4	6.9	7.5
	Obesity	31.3	12.1	19.2		Obesity	15.8	7.4	8.4
**156–157**	Underweight	13.5	5.8	7.7	**182–183**	Underweight	12.7	7.0	5.6
	Normal weight	19.2	7.2	12.0		Normal weight	11.7	6.2	5.5
	Overweight	23.7	8.9	14.7		Overweight	12.5	7.3	5.2
	Obesity	29.2	10.8	18.4		Obesity	14.1	6.1	8.1
**158–159**	Underweight	14.2	5.5	8.7	**184–185**	Underweight	3.0	3.0	0
	Normal weight	17.8	6.9	10.9		Normal weight	9.7	5.7	4.0
	Overweight	22.7	8.5	14.1		Overweight	14.6	7.1	7.5
	Obesity	26.8	10.8	16.0		Obesity	20.1	8.6	11.5
**160–161**	Underweight	13.6	5.4	8.2	**186–187**	Underweight	41.7	33.3	8.3
	Normal weight	16.5	7.0	9.5		Normal weight	11.9	7.7	4.2
	Overweight	20.0	7.7	12.4		Overweight	14.7	8.4	6.3
	Obesity	24.7	9.5	15.2		Obesity	16.3	11.2	5.0
**162–163**	Underweight	11.5	5.3	6.1	**188–189**	Underweight	25.0	0	25.0
	Normal weight	15.3	6.6	8.7		Normal weight	20.9	13.4	7.5
	Overweight	18.4	7.4	11.0		Overweight	7.1	7.1	0
	Obesity	23.5	8.9	14.6		Obesity	33.3	33.3	0
**164–165**	Underweight	11.9	5.5	6.4	**≥190**	Underweight	-	0	0
	Normal weight	14.4	6.5	7.9		Normal weight	13.9	13.9	0
	Overweight	17.9	7.6	10.3		Overweight	16.7	8.3	8.3
	Obesity	22.1	8.8	13.3		Obesity	12.5	0	12.5

^1^Proportions calculated for the distribution in specified BMI-class for each maternal height category

### Univariate and multiple regression analyses and population attributable proportions

**Fig 3:**
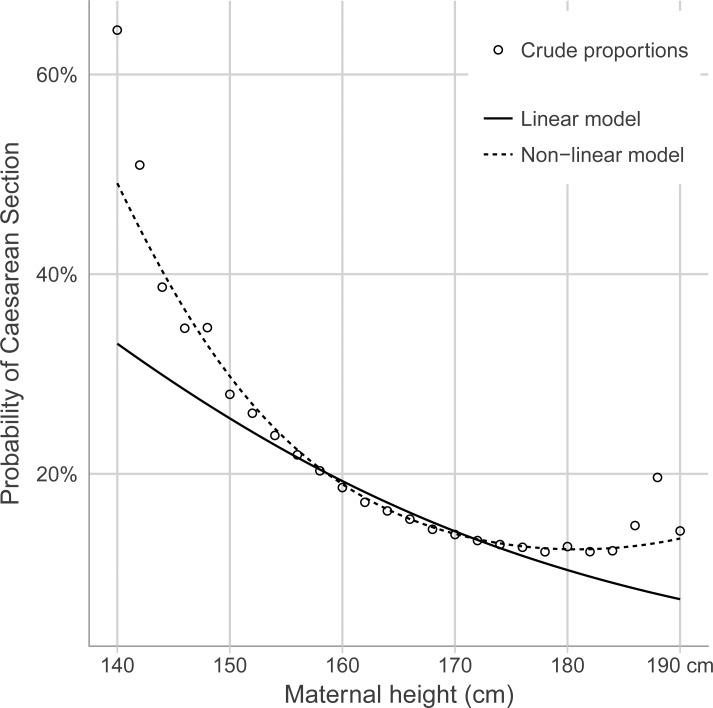
Probability of caesarean section in relation to maternal height (cm) estimated by a linear and a non-linear model. Crude proportions are plotted in the figure (unfilled circles).

In univariate logistic regression analyses, the crude odds ratios (COR) and their 95% confidence intervals (95% CI) for CS in relation to vaginal delivery for specified categories of maternal height were calculated (Table 5). In these analyses, maternal height 166–167 cm was selected as the reference category (Table 5). Table 5 demonstrates a non-linear relation for decrease in risk for CS by maternal height, and the relationship was further investigated by estimating the probability of CS at a given maternal height using logistic regression. For example, maternal height 152–153 cm presented an almost doubled risk of CS (COR 1.93; 95% CI 1.82–2.04), whereas maternal height 178–179 cm presented the lowest risk of CS (COR 0.76; 95% CI 0.71–0.81). The COR for emergency CS was also lowest in the maternal height category of 178–179 cm (COR 0.65; 95% CI 0.59–0.72) for women born in Sweden, whereas the maternal height category of 182–183 cm demonstrated the lowest risk of emergency CS for women born outside Sweden (Table 5). In Fig 3 the relationship between maternal height and CS was further explored using both a linear effect for maternal height and the use of a second degree polynomial for maternal height. The non-linearity motivated the use of a second order term for maternal height in the following multiple logistic regression analyses. Table 6 presents mean birth weight in grams, proportions of birth weight 4500 grams or more in relation to maternal height categories and country of birth. Table 6 also presents odds ratios and their 95% confidence intervals for risk of CS adjusted by birth weight of 4500 grams or more for each maternal height category.

**Table 5:** Crude odds ratios (COR) and their 95% confidence intervals (95% CI) for caesarean section (CS) in relation to vaginal delivery in logistic regression analyses for specified categories.

Maternal height (cm)	Caesarean section (N = 570,445)		CS Born in Sweden (n = 417,495)		CS Born outside Sweden (n = 124,251)		Emergency CS Born in Sweden^a^ (n = 388,050)		Emergency CS Born outside Sweden^a^ (n = 115,438)	
	COR	95% CI	COR	95% CI	COR	95% CI	COR	95% CI	COR	95% CI
**140–141**	9.91	5.38–18.26	10.83	2.70–43.33	11.22	5.40–23.34	3.22	0.33–31.03	6.68	2.58–17.27
**142–143**	5.68	3.88–8.29	4.69	2.23–9.87	5.72	3.62–9.04	3.87	1.50–9.99	4.41	2.45–7.96
**144–145**	3.45	2.75–4.32	5.11	3.17–8.23	3.39	2.58–4.46	5.26	3.00–9.20	3.03	2.14–4.29
**146–147**	2.89	2.48–3.37	3.64	2.73–4.86	2.88	2.36–3.51	3.09	2.12–4.48	3.30	2.61–4.18
**148–149**	2.90	2.58–3.26	4.03	3.21–5.07	2.85	2.46–3.31	4.02	3.05–5.29	3.25	2.72–3.88
**150–151**	2.12	1.98–2.28	2.56	2.28–2.88	2.09	1.89–2.32	2.75	2.39–3.16	2.35	2.07–2.66
**152–153**	1.93	1.82–2.04	2.12	1.96–2.31	1.94	1.77–2.12	2.43	2.20–2.68	2.25	2.01–2.51
**154–155**	1.71	1.63–1.80	1.92	1.79–2.05	1.67	1.54–1.82	2.11	1.95–2.29	1.87	1.68–2.07
**156–157**	1.53	1.47–1.60	1.65	1.57–1.74	1.49	1.38–1.62	1.86	1.74–1.98	1.64	1.48–1.81
**158–159**	1.39	1.34–1.45	1.44	1.38–1.51	1.40	1.29–1.51	1.56	1.48–1.66	1.54	1.40–1.70
**160–161**	1.25	1.21–1.30	1.29	1.24–1.35	1.23	1.14–1.33	1.38	1.31–1.45	1.32	1.20–1.45
**162–163**	1.13	1.09–1.17	1.16	1.12–1.21	1.11	1.02–1.19	1.22	1.16–1.28	1.15	1.05–1.27
**164–165**	1.06	1.03–1.10	1.05	1.02–1.10	1.12	1.04–1.21	1.07	1.02–1.13	1.15	1.04–1.26
**166–167**	**1.00**	-	**1.00**	-	**1.00**	-	**1.00**	-	**1.00**	-
**168–169**	0.92	0.89–0.96	0.92	0.88–0.96	0.92	0.84–0.997	0.90	0.85–0.95	0.87	0.78–0.97
**170–171**	0.88	0.85–0.91	0.88	0.84–0.91	0.92	0.83–0.998	0.83	0.79–0.88	0.87	0.78–0.98
**172–173**	0.84	0.81–0.88	0.83	0.79–0.87	0.94	0.84–1.04	0.76	0.71–0.80	0.88	0.76–1.00
**174–175**	0.81	0.77–0.85	0.80	0.76–0.83	0.88	0.78–0.992	0.71	0.67–0.76	0.86	0.74–1.01
**176–177**	0.79	0.75–0.84	0.78	0.73–0.83	0.90	0.77–1.06	0.70	0.65–0.76	0.83	0.67–1.04
**178–179**	0.76	0.71–0.81	0.74	0.69–0.80	0.93	0.79–1.12	0.65	0.59–0.72	0.87	0.68–1.12
**180–181**	0.80	0.74–0.86	0.81	0.74–0.88	0.69	0.53–0.90	0.71	0.63–0.79	0.63	0.43–0.90
**182–183**	0.76	0.67–0.86	0.78	0.68–0.89	0.66	0.43–1.01	0.66	0.55–0.80	0.42	0.21–0.84
**184–185**	0.77	0.63–0.93	0.77	0.63–0.94	0.83	0.44–1.57	0.65	0.49–0.87	0.67	0.27–1.67
**186–187**	0.95	0.73–1.24	0.92	0.69–1.23	1.27	0.59–2.74	0.58	0.36–0.91	1.13	0.40–3.19
**188–189**	1.34	0.83–2.14	1.32	0.79–2.18	0.65	0.83–5.17	0.62	0.25–1.54	1.17	0.14–9.22
**≥190**	0.91	0.46–1.79	0.69	0.29–1.62	1.96	0.53–7.25	0.62	0.19–1.99	-	-

^a^Women with emergency caesarean section and vaginal delivery (reference) included in analyses.

**Table 6:** Singleton birth weight (grams; g) in relation to maternal height for all women, for women born in Sweden (S) and for women born outside Sweden (OS) for specified outcomes. Adjusted odds ratio and 95% confidence interval for risk of caesarean section^1^ (CS).

Maternal height (cm)	Mean birth weight; g (MBW)	S		OS		Birth weight (BW) >4500 g % (n)		S	OS	CS (n = 565,498)	
		MBW g	Min^2^-max^3^ g	MBW g	Min^2^-max^3^ g			BW ≥4500 g %	BW ≥4500 g %	AOR^1^	95% CI
**140–141**	3020	2976	2610–3975	3083	2330–3930	0	0	0	0	9.82	5.31–18.17
**142–143**	3127	3114	1755–4090	3155	1100–4615	1.5	1	0	1.3	5.79	3.97–8.46
**144–145**	3169	3083	1375–4190	3215	642–4575	0.5	1	0	1.3	3.51	2.80–4.41
**146–147**	3165	3120	670–4880	3185	805–4680	0.7	3	1.6	0.4	2.92	2.50–3.41
**148–149**	3209	3180	1060–4655	3219	395–4980	0.7	6	0.7	0.7	2.96	2.63–3.32
**150–151**	3249	3242	622–4880	3257	485–5120	0.9	26	1.1	0.8	2.16	2.02–2.32
**152–153**	3290	3305	400–5032	3282	345–5368	0.9	46	0.7	0.9	1.98	1.87–2.09
**154–155**	3317	3335	404–5880	3302	470–5565	1.4	120	1.4	1.2	1.74	1.66–1.83
**156–157**	3368	3379	378–6280	3356	334–5700	1.5	203	1.5	1.5	1.55	1.49–1.62
**158–159**	3395	3410	326–5734	3372	412–6040	1.6	326	1.7	1.6	1.42	1.36–1.47
**160–161**	3443	3461	300–5880	3407	335–6200	2.2	755	2.3	1.9	1.27	1.22–1.31
**162–163**	3477	3492	300–5800	3434	435–5712	2.5	1012	2.6	2.0	1.14	1.10–1.18
**164–165**	3515	3531	330–6640	3463	300–6100	3.0	1459	3.3	2.2	1.06	1.03–1.10
**166–167**	3550	3562	300–5890	3500	348–6240	3.3	1174	3.5	2.8	**1.00**	
**168–169**	3579	3589	350–6270	3525	396–6020	4.0	1715	4.1	2.9	0.92	0.89–0.95
**170–171**	3614	3625	387–6180	3550	370–6205	4.4	1729	4.5	3.4	0.88	0.84–0.91
**172–173**	3646	3654	300–5915	3589	442–5995	5.4	1567	5.4	4.2	0.83	0.79–0.86
**174–175**	3676	3682	360–6100	3631	300–5545	6.0	1241	5.8	5.3	0.80	0.76–0.83
**176–177**	3702	3708	300–6050	3650	440–5560	6.4	706	6.6	5.0	0.77	0.73–0.82
**178–179**	3727	3732	350–5940	3670	1000–5725	7.1	574	7.3	5.3	0.74	0.69–0.79
**180–181**	3761	3773	625–6200	3660	1208–5470	9.3	438	9.2	6.9	0.77	0.71–0.83
**182–183**	3806	3810	560–5654	3756	1350–5270	10.1	175	10.0	6.1	0.72	0.64–0.82
**184–185**	3824	3825	305–5815	3817	1535–5830	9.9	67	11.0	8.1	0.73	0.60–0.89
**186–187**	3836	3878	465–5450	3524	650–4770	12.2	36	13.9	2.2	0.90	0.69–1.17
**188–189**	3786	3794	1435–4960	3763	2635–4835	10.3	8	9.4	10.0	1.30	0.81–2.08
**≥190**	3867	3920	2375–5270	3757	2535–4144	16.3	7	20.8	0	0.75	0.37–1.52
***All***	***3534***	***3565***	***300–6640***	***3438***	***300–6240***	***3*.*6***	***20*,*481***	***3*.*9***	***2*.*3***		

^1^Multiple regression model with adjustment for birth weight 4500 grams or more (ref. group birth weight <4500 grams)

^2^Birth weight minimum value

^3^Birth weight maximum value

**Fig 4:**
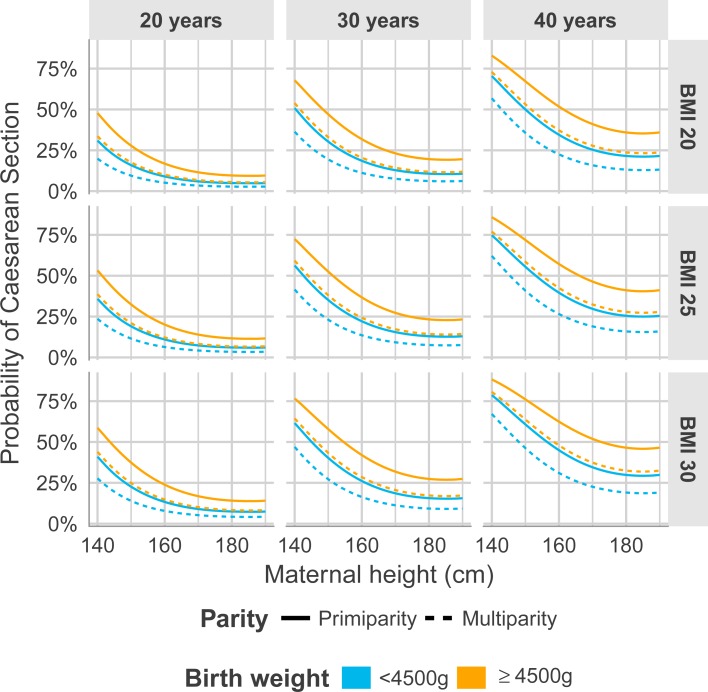
Predicted probability of CS for the panels of specific age in years (20, 30, 40), specific BMI (20, 25, 30), birth weight less than 4500 g or 4500 g or more, and parity represented by the categories primiparity and multiparity.

**Fig 5:**
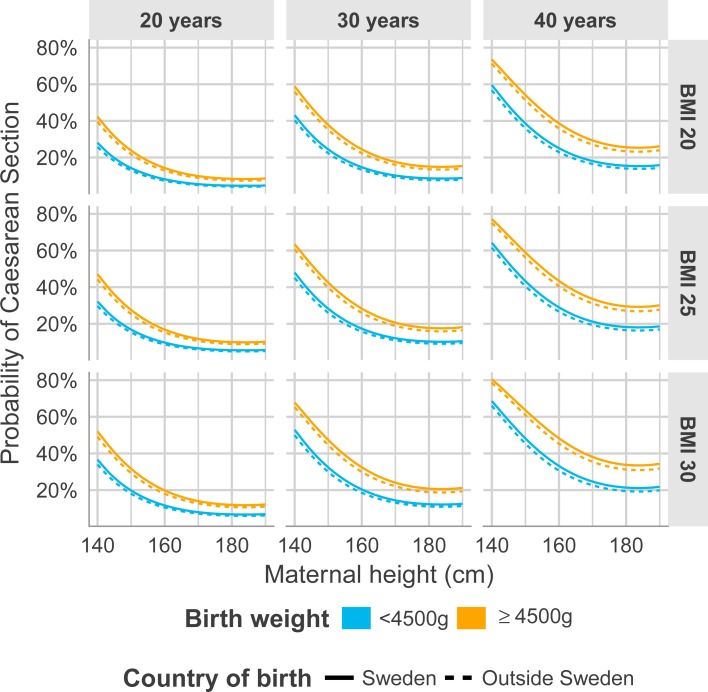
Predicted probability of CS for the panels of specific age in years (20, 30, 40), specific BMI (20, 25, 30), birth weight less than 4500 g or 4500 g or more, and country of birth represented by the categories born in Sweden and born outside Sweden.

Table 7 presents univariate logistic regression analyses for the risk of CS and emergency CS in relation to the continuous variables maternal height (cm), BMI (kg/m^2^), age (yrs) and gestational age (days) and the categorical variables parity, country of birth and birth weight grouped. In the multiple regression model (Table 7) including all the variables from the univariate analyses, a second degree term of maternal height was added in order to capture the non-linearity. The effect levels for maternal height are comparable in dimension with the effect of BMI and age on risk of CS. The corresponding decrease of risk in relation to increase in maternal height for emergency CS was slightly lower (Table 7). To be noted in the multiple regression models was that country of birth diminished as an explanatory variable for increased risk of CS (Table 7). In order to visually interpret the multiple regression model the predicted probability of CS was estimated from the models at specified ages (i.e. 20, 30 and 40 years), specified BMI (i.e. 20, 25, and 30 kg/m^2^), birth weight (i.e. <4500 g and ≥4500 g) and parity (primiparous and multiparous) (Fig 4). A corresponding figure was created showing the predicted probability of CS estimated from the models at specified ages (i.e. 20, 30 and 40 years), specified BMI (i.e. 20, 25, and 30 kg/m^2^), birth weight (i.e. <4500 g and ≥4500 g) and country of birth (born in Sweden and born outside Sweden) (Fig 5). The relative risk (RR) for women with a height of 159 cm or below to undergo CS in relation to women 160 cm or taller was 1.50 (95% CI 1.48–1.53) with a population attributable proportion of 6.8%, meaning that 6.8% of CS could be explained by shorter maternal stature. When evaluating the corresponding risk and PAP for women with a maximum height of 155 cm in relation to taller women (156 cm or higher) the RR and its 95% CI was relatively higher (RR = 1.66; 95% CI 1.63–1.70) with a PAP of 3.4%.

**Table 7:** Univariate and multiple logistic regression analyses, with crude odds ratios (COR) and adjusted odds ratios (AOR) and 95% confidence intervals (CI) for caesarean section in relation to vaginal delivery for specified explanatory variables.

Continuous and categorised variables	Caesarean section (CS) Univariate logistic regression			Caesarean section (CS) Multiple logistic regression			Emergency (CS) Univariate logistic regression			Emergency (CS) Multiple logistic regression		
	COR	95% CI	n^a^	AOR	95% CI	n^a^	COR	95% CI	n^a^	AOR	95% CI	n^a^
Maternal height (cm)	0.964	0.963–0.965	570,418	0.671	0.643–0.643	521,918	0.953	0.952–0.955	345,431	0.717	0.670–0.767	323,435
Maternal height^2^ (cm^2^)				1.001	1.001–1.001	521,918				1.00	1.00–1.00	323,435
Maternal BMI (kg/m^2^)	1.047	1.046–1.049	564,204	1.045	1.043–1.046	521,918	1.058	1.055–1.060	343,002	1.062	1.059–1.064	323,435
Maternal age (years)	1.067	1.066–1.069	581,648	1.087	1.085–1.089	521,918	1.037	1.034–1.039	345,365	1.075	1.073–1.078	323,435
Gestational age (days)	0.976	0.975–0.976	571,855	0.976	0.975–0.976	521,918	0.982	0.982–0.983	336,108	0.982	0.981–0.983	323,435
Parity												
*Multiparity*	1.00			1.00			1.00			1.00		
*Primiparity*	1.258	1.240–1.275	573,543	1.809	1.779–1.838	521,918	2.317	2.263–2.372	344,941	3.429	3.338–3.523	323,435
Country of birth												
*Sweden*	1.00			1.00			1.00			1.00		
*Outside Sweden*	1.094	1.076–1.113	549,467	0.922	0.904–0.939	521,918	1.113	1.103–1.164	338,607	0.959	0.930–0.989	323,435
Birth weight (grams)												
*<4500*	1.00			1.00			1.00			1.00		
*≥4500*	1.636	1.583–1.691	576,119	2.044	1.971–2.119	521,918	1.948	1.855–2.045	341,643	2.815	2.670–2.968	323,435

^a^Number included in analyses

## Discussion

The main finding of this study was that maternal height exerts an effect on the risk of CS, with decreasing risk of CS with increasing maternal height. This effect remained after adjustment for other known risk factors for CS such as maternal age, BMI, gestational age, parity, high birth weight and country of birth. The effect of maternal height was found to be non-linear with the greatest implications for women of short stature. The inverse effect of increasing maternal height on risk of CS can be compared with the well-known risk factors of maternal BMI and maternal age on risk for CS. Maternal height is evidently a non-modifiable factor, so are also maternal age and maternal BMI during pregnancy. Maternal age and BMI are considered significant factors in clinical management and in counselling during pregnancy and delivery. Our findings show that maternal height is also an important factor to consider. Taller stature exerts a protective effect on risk for CS, and that can be more emphasised in counselling on mode of birth in order to promote vaginal birth and lower the CS rate. Body mass index is a well-known risk factor for CS with increasing risks with increasing BMI [46]. For each maternal height category there was a clear pattern demonstrating increasing risk for CS with increasing BMI-class. Pregnant women with maternal height less than 156 cm demonstrated a higher BMI, i.e. overweight, in relation to women with taller maternal height. This combination of background risk factors for caesarean section most probably contributed to increased risks for CS for this shorter maternal height category. In a recent published study, where short maternal stature was defined as maternal height less than 156 cm, the authors concluded that “The combination of maternal short stature and overweight was associated with a more than threefold risk of subsequent hypoxic ischemic encephalopathy” [47].

There was an absolute increased risk of CS among women born outside Sweden in comparison to women born in Sweden (17.3% vs. 16.0%), but in the multiple regression model country of birth outside Sweden diminished as a risk factor for increased risk of CS after adjustment for factors such as maternal height, BMI, maternal age, gestational age, parity and high birth weight. In this study we did not undertake sub-analyses related to specific countries or geographic areas of birth, which might have demonstrated differences in relation to country of origin. However, we plan to investigate this further in a future paper.

When calculating the population attributable proportion for women with maternal height below 160 cm, maternal height could explain around 7% of cases undergoing CS, thus constituting a significant risk factor for CS. Although height is non-modifiable, clinicians should increasingly consider short maternal stature as a significant risk factor for CS. However, the mechanisms by which short stature increased the risk of CS may be partly confounded by the fact that women, who were themselves exposed to growth retardation during fetal life, demonstrate an increased risk of metabolic syndrome as adults, including severe pre-eclampsia, which is a significant risk factor for CS [48, 49]. Further, these women may produce small for gestational age babies, thus transferring the increased risk of CS not through genome-mediated smallness, but through epigenetic mechanisms [48–50]. Regarding gestational age, our study confirms previous findings of increasing maternal height demonstrating a significant correlation with increasing gestational age [15].

### Methodological considerations

This study demonstrates a number of methodological strengths. The study period included six years of data, thus establishing a large sample size with sufficient power for the research questions under study, indicated by overall narrow confidence intervals. During the first two years of the study period data were registered manually by midwives in antenatal care, into the then called Swedish Maternal Health Care Register (MHCR). We have previously been able to demonstrate that data in the MHCR are of high quality [41]. Since 2013, MHCR has been part of the Swedish Pregnancy Register, and data have increasingly been imported through automatic electronic transfer from medical records. Accordingly, almost all variables in the current dataset from 2013 to 2016 were electronically transferred. The coverage of the data from 2011 to 2016 increased during the study period, mostly due to the automatic electronic transfer of data. We have previously published results from the MHCR and the SPR, demonstrating that data in these quality registers do not deviate substantially from the mandatory Swedish Medical Birth Register covering more than 99% of all births in Sweden [51]. A possible limitation of this study may be the self-reported nature of some variables, particularly maternal weight, and perhaps to a lesser extent parity and country of birth. Another limitation of the study is the non-adjustment for other significant maternal conditions such as for example hypertensive disorders during pregnancy since those diagnoses were not accessible in the registers for the whole study period. In addition, we cannot exclude the role of clinician choice to perform caesarean section in women with shorter stature in the absence of other risk factors. The variable maternal age was established using the birth date of the woman and the delivery date, where maternal birth date was obtained automatically from the Swedish Population Registry. Calculation of gestational age was based primarily on second trimester ultrasound dating examinations, but also first trimester ultrasounds, for more than 99% of the women in the sample.

## Conclusions

Maternal height exerts an absolute effect on the risk of CS, with decreasing risk of CS with increasing maternal height. Although a higher proportion of women born outside Sweden had a CS in comparison with women born in Sweden, country of birth diminished as a risk factor in the multiple regression model after adjustment for other established risk factors for CS. Body mass index and maternal age are factors already used in clinical assessments related to birth, and maternal height should increasingly enjoy a similar status in these considerations. Moreover, when health professionals are counselling pregnant women, taller stature should be more emphasized as a positive indicator for successful vaginal birth in order to increase pregnant women’s confidence in giving birth vaginally, with possible positive impacts for lowering CS rates.
